# Pulmonary Sclerosing Pneumocytomas Mimicking Lung Cancer

**DOI:** 10.7759/cureus.37395

**Published:** 2023-04-10

**Authors:** Daniella Lizarraga Madrigal, Manuel Cabrera Charleston, Asad Khan, George Eapen, Neda Kalhor, Qiong Gan, Horiana Grosu

**Affiliations:** 1 Internal Medicine, Instituto Tecnologico y de Estudios Superiores de Monterrey, Monterrey, MEX; 2 Internal Medicine, Instituto Tecnológico y de Estudios Superiores de Monterrey, Monterrey, MEX; 3 Pulmonology, Monroe Dunaway (MD) Anderson Cancer Center, Houston, USA; 4 Pulmonary Medicine, Monroe Dunaway (MD) Anderson Cancer Center, Houston, USA; 5 Pathology, Monroe Dunaway (MD) Anderson Cancer Center, Houston, USA; 6 Pathology, University of Texas Monroe Dunaway (MD) Anderson Cancer Center, Houston, USA

**Keywords:** lung mass, robotic bronchoscopy, lung cancer, pulmonary sclerosing pneumocytomas, pulmonary nodule

## Abstract

Pulmonary sclerosing pneumocytomas are benign tumors. These tumors are often found incidentally and can be challenging to distinguish from lung malignancies. Here, we describe the case of a 31-year-old woman who presented with an incidental finding of a lung nodule in the lingula. She was asymptomatic and had no history of cancer. Positron emission tomography showed [18F] fluorodeoxyglucose (FDG) uptake in the nodule but no FDG-avid mediastinal lymphadenopathy. In view of these findings, a bronchoscopy was performed, and biopsy samples were taken. The final pathological diagnosis revealed a sclerosing pneumocytoma.

## Introduction

Pulmonary sclerosing pneumocytoma is a low-prevalence benign lung tumor. It predominantly affects middle-aged Asian women. It is commonly found incidentally by X-ray or CT imaging of the chest and may be mistaken for adenocarcinoma and other solid tumor malignancies [1]. We present a case of a 31-year-old woman who presented to our clinic for evaluation of an incidentally found, asymptomatic lung nodule that showed [18F] fluorodeoxyglucose (FDG) uptake on positron emission tomography (PET), resulting in a suspicion for malignancy. The tumor was determined to be a sclerosing pneumocytoma based on pathologic analysis. 

## Case presentation

**Figure 1:**
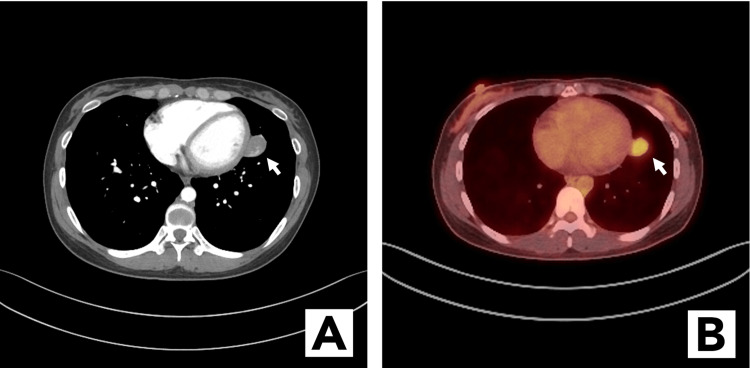
(A) CT showing a nodule in the lingula abutting the major fissure. (B) PET-CT image with FDG-avid showing uptake in the nodule. PET, positron emission tomography; FDG, fluorodeoxyglucose

The patient was a 31-year-old Asian woman who presented to our institution for evaluation of an incidentally found left lung nodule. The patient, a lifelong nonsmoker, had recently had mild coronavirus disease 2019 (COVID-19) and influenza, resulting in a prolonged cough that lasted over 6 weeks. As part of the evaluation for the cough, by her primary care physician, she had a chest X-ray followed by a CT scan of the chest, which revealed a 2.7-cm well-circumscribed nodule in the lingula abutting the major fissure (Figure 1A). On our evaluation, the patient was asymptomatic with no other respiratory concerns, except for cough. She reported no weight loss, hemoptysis, or shortness of breath. For further evaluation of the lung nodule, she underwent a PET scan, which showed FDG uptake in the mass (Figure 1B). There was no associated FDG-avid hilar or mediastinal adenopathy. 

**Figure 2:**
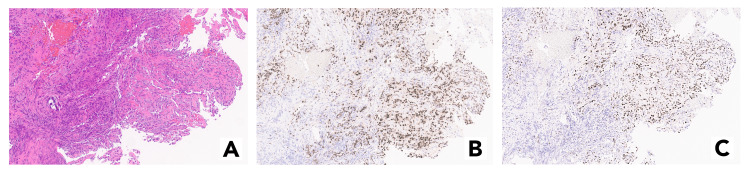
(A) High magnification view shows a tumor composed of epithelioid cells with focal papillary features (red arrow). Focal calcification and hemorrhage are noted (blue and yellow, respectively). Tumor cells lining the papillae appear cuboidal and slightly smaller. No mitosis is identified. (B) TTF-1 expression is present in majority of the tumor cells. (C) Progesterone receptor expression is present in ~60% of the tumor cells. TTF-1, thyroid transcription factor-1

The patient underwent robotic bronchoscopy with fine-needle biopsy of the nodule. Cytologic evaluation of cell block sections from fine-needle aspiration of the nodule showed papillary fragments with sclerotic stroma lined by cuboidal cells, in a background of round polygonal epithelial cells with abundant cytoplasm (Figure 2A). Immunoperoxidase staining showed that the tumor cells were diffusely positive for thyroid transcription factor-1 (TTF-1) and only patchy positive for pankeratin, and negative for chromogranin (Figure 2B). Progesterone receptor expression was noted in 70% of the tumor cells (Figure 2C). Based on the tumor’s morphological features and immune profile, the diagnosis of sclerosing pneumocytoma was made. The patient is currently scheduled to undergo resection of the left lung via robotic-assisted thoracic surgery. 

## Discussion

Pulmonary sclerosing pneumocytoma is a low-prevalence, benign lung tumor. Before the publication of the 2015 World Health Organization (WHO) Classification of Tumors, it was known as sclerosing hemangioma, but it is now classified as a pulmonary adenoma [2].[This tumor represents a diagnostic challenge because of the diversity of reported histopathological findings associated with it. It was first described in 1956 as a neoplasia with a vascular endothelial cell origin [3].

Our patient was typical of patients affected by sclerosing pneumocytoma, which predominantly affects middle-aged Asian women who are nonsmokers. As was the case for our patient, most sclerosing pneumocytomas are asymptomatic and found incidentally on chest CT scans or X-rays conducted for other reasons. When symptoms are present, they can include a dry cough, chest pain, hemoptysis, or shortness of breath [4]. Chest CT typically shows well-circumscribed, oval lung nodules with smooth outlines. On PETCT, the lung nodule often shows uptake of FDG, which is usually indicative of malignancy; thus, these tumors are often misdiagnosed as lung cancers [5].

As observed in the histopathology findings for this case, sclerosing pneumocytomas are characteristically composed of two cell types: cuboidal or surface lining cells, and round or stromal cells, both of which stain positive for TTF-1 [6]. Additionally, the surface cells typically express epithelial membrane antigen, cytokeratin, and surfactant apoprotein A [1]. These characteristics suggest, according to a study published by Chan et al. [7], that these tumors are derived from either primitive respiratory epithelium or incompletely differentiated type II pneumocytes. As revealed in a 2020 study by Maleki et al. [8], foamy macrophages, nuclear inclusions, and hyalinized fragments are commonly observed cytomorphologic features of sclerosing pneumocytoma. Similarly, the nuclei may occasionally show pleomorphism, hyperchromasia, and prominent nucleoli.

Although no official guidelines for the management of sclerosing pneumocytoma have been published yet, a study conducted by Park et al. [9] compared lobectomy to limited resection for the treatment of these tumors. In that study, all patients, regardless of the procedure they underwent, were free of local recurrence and distant metastasis during the follow-up period.

## Conclusions

This case highlights the diagnostic challenge represented by the diverse reported radiological and histopathological findings associated with sclerosing pneumocytoma. Clinicians should be aware of the possibility of a false-positive finding of malignancy on FDG PET‐CT when evaluating lung tumors. 
